# Counting mitoses: SI(ze) matters!

**DOI:** 10.1038/s41379-021-00825-7

**Published:** 2021-06-02

**Authors:** Ian A. Cree, Puay Hoon Tan, William D. Travis, Pieter Wesseling, Yukako Yagi, Valerie A. White, Dilani Lokuhetty, Richard A. Scolyer

**Affiliations:** 1 International Agency for Research on Cancer (IARC), World Health Organization, Lyon, France; 2 Division of Pathology, Singapore General Hospital, Singapore, Singapore; 3 Department of Pathology, Memorial Sloan Kettering Cancer Center, New York, NY USA; 4 Department of Pathology, Amsterdam University Medical Centers/VUmc, Amsterdam, The Netherlands; 5 Princess Máxima Center for Pediatric Oncology, Utrecht, The Netherlands; 6 Department of Pathology, Faculty of Medicine, University of Colombo, Colombo, Sri Lanka; 7 Melanoma Institute Australia and Faculty of Medicine and Health, The University of Sydney, Tissue Pathology and Diagnostic Oncology, Royal Prince Alfred Hospital, and NSW Health Pathology, Sydney, NSW Australia

**Keywords:** Pathology, Outcomes research

## Abstract

Mitoses are often assessed by pathologists to assist the diagnosis of cancer, and to grade malignancy, informing prognosis. Historically, this has been done by expressing the number of mitoses per *n* high power fields (HPFs), ignoring the fact that microscope fields may differ substantially, even at the same high power (×400) magnification. Despite a requirement to define HPF size in scientific papers, many authors fail to address this issue adequately. The problem is compounded by the switch to digital pathology systems, where ×400 equivalent fields are rectangular and also vary in the area displayed. The potential for error is considerable, and at times this may affect patient care. This is easily solved by the use of standardized international (SI) units. We, therefore, recommend that features such as mitoses are always counted per mm^2^, with an indication of the area to be counted and the method used (usually “hotspot” or “average”) to obtain the results.

## Background

Mitoses and other features, such as the degree of nuclear atypia, necrosis, vascularity, and invasion are evaluated by pathologists in making a diagnosis of cancer, and to grade malignancy, informing prognosis. In many publications, mitotic activity is given as a mitotic count, expressed as the number of mitoses per high-power field (HPF), or per 10 or 50 HPFs. High power is usually taken as ×400 overall magnification, where a ×40 objective is paired with a ×10 eyepiece. Unfortunately, different combinations of microscopes and lenses result in widely variable actual areas of the high-power field. This may impart significant measurement errors amongst observers using different microscopes. A number of tumor types in which the diagnosis may be affected is shown in Table 1, based on searches of the current fourth and fifth edition WHO classification of tumors.

**Table 1:** Examples of tumor types for which diagnosis or prognosis requires re-assessment of mitoses in the current fifth edition WHO Classification of Tumors due to previous use of undefined HPF.

WHO Classification of Tumors volume	Tumor type
Female genital tract [7]	Smooth muscle tumors, uterine
Female genital tract [7]	Smooth muscle tumors, extrauterine
Soft tissue and bone [8]	Solitary fibrous tumor
Female genital tract [7]	Clear cell carcinoma of the uterus
Breast [15]	Phyllodes tumors
Soft tissue and bone [8]	Chondrosarcomas
Female genital tract [7]	Low-grade serous carcinoma of the ovary

This list is not exhaustive but is based on a search of the website (https://tumourclassification.iarc.who.int).

**Fig. 1:**
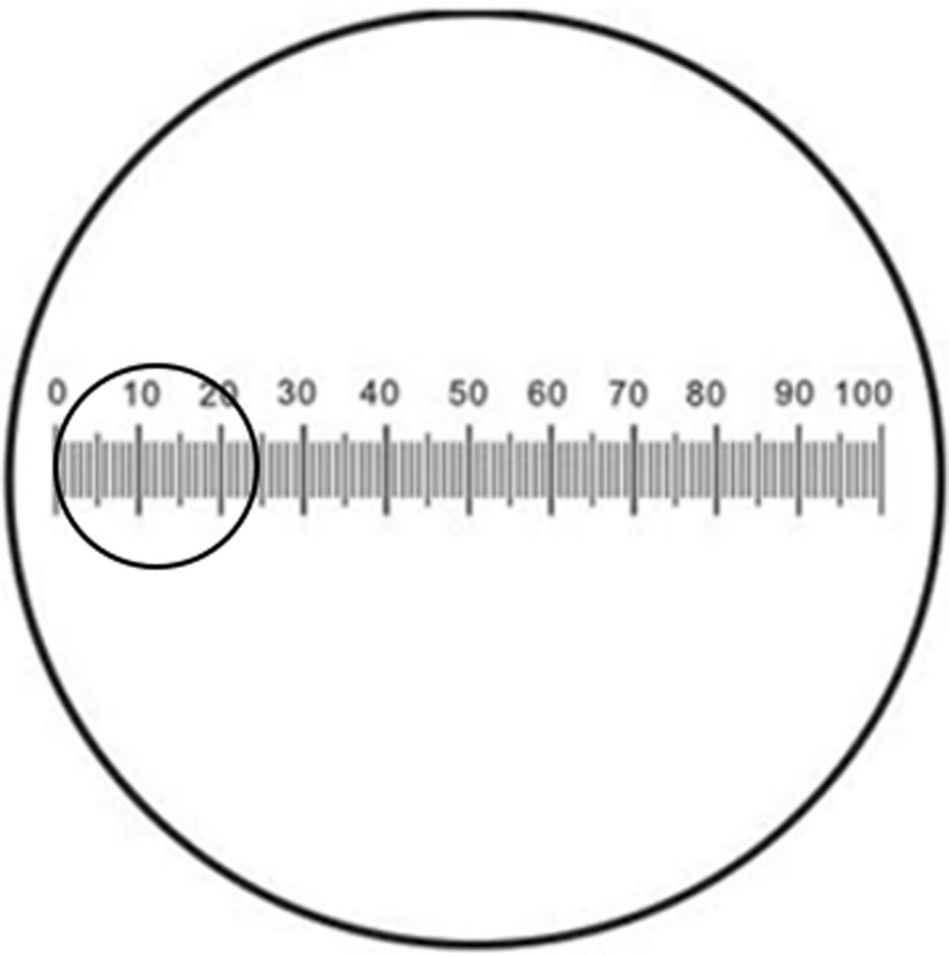
Diagram of a typical 400× microscope field  (small circle, 0.24 mm^2^) superimposed on a simple slide micrometer scale, permitting measurement of the diameter of the field of view of a microscope with an objective and eyepiece combination. High power fields (HPF) conventionally use the ×40 objective, giving an overall magnification of ×400 with a 10× eyepiece, or ×500 with a 12.5× eyepiece. The area is calculated by *πr*^2^, where “*r*” is the radius (half the diameter) of the field of view.

It is accepted that the best practice is to at least define the size of these fields in scientific publications [1], but unfortunately, journals have not enforced the requirement that the size of the field used for a study be specified. It is our view that the use of HPF should be abandoned completely moving forward and replaced by standardized international (SI) units, which are the required units of work in medicine and science, as the term HPF cannot be standardized. The use of millimeters squared (mm^2^) gives an appropriately standardized unit for area measurement and mitoses per mm^2^ are easily calculated (Fig. 1). Furthermore, the data obtained are independent of the microscope and magnification used and the results obtained in different parts of the world would therefore become more comparable.

## The extent of the issue

The problems caused by the use of HPF as a unit of area measurement have long been recognized, particularly in publications from the 1980s and 1990s [1–6]. However, these have been largely ignored in some parts of the discipline, and it is still common to find textbooks and even the fourth edition WHO Blue Books [7, 8] advising pathologists to determine malignancy on the basis of a number of mitoses per HPF which are not defined. The consequence is that where one pathologist may call a particular tumor malignant, the other may call it benign, simply based on the microscope that they are using at the time. For many tumor types, the papers on which these measurements are based are more than 10 years old and pre-date modern digital pathology. Those published prior to 2000 are likely to have used microscopes with small fields of view, as field diameters/areas have increased with the use of improved lens technology over recent decades. In addition, some microscopes have wide field eyepieces with a larger field of view than those with standard eyepieces [9]. With microscopes having wide field eyepieces, fewer fields should be counted to reach the equivalent of 1 mm^2^ than those with standard eyepieces. Adjustments may also need to be made if the microscope has any additional magnifier such as a magnification changer or an intermediate attachment [9].

The first report of variation in HPFs leading to potentially incorrect diagnosis was made by Ellis and Whitehead in 1981 [2] in the context of deciding the malignancy of uterine smooth muscle tumors. To illustrate the implications of this in the counting of mitoses, they used a hypothetical tumor containing 500 cells/mm^2^, and one mitotic figure per 100 cells, which should produce 5 mitoses/mm^2^. They measured this tumor using HPF of 0.071 mm^2^ and 0.414 mm^2^. In this instance, the microscope with the smallest field of view will only produce an average of 3.55 mitoses per 10 HPF, while the largest will produce 20.7 mitoses per 10 HPF. They comment, “this is a nearly sixfold variation, representing the difference between an obviously benign tumor and one that most pathologists would diagnose as malignant”.

As a result of the papers published in the last century, many pathologists decided to define the size of their HPF and/or adopted counts per mm^2^ for their research and practice, particularly for tumors of the lung [9], breast [10–12], and for cutaneous melanomas [4, 5, 13]. Conversion tables have been provided for breast cancer and haematolymphoid tumor grading for many years, and are included within the WHO classification of tumors [14, 15]. The standardization of counting mitoses per 2 mm^2^ has been established for neuroendocrine lung tumors in WHO classifications of Lung Tumors for over 20 years, beginning in 1999, in the second edition. However, this was not the case in other volumes. The fifth edition WHO Classification of Tumors is now publishing all mitotic counts as “mitoses/mm^2^” with additional details provided regarding the area recommended for counting, and the counting method to be used.

## Mitotic activity: definition of terms

The problem is particularly important for the assessment of cell proliferation. Cell division by mitosis is recognizable in histological sections and has long provided an important means of assessing the proliferation of tumors. There are essentially three options for the measurement of cell proliferation by mitosis:

*Mitotic count* is a simple density measurement, now usually expressed as the number of mitoses per mm^2^ rather than per HPF. It still takes no account of cell size, the presence of intratumoral stroma separating tumor cells which impacts tumor cell density, the number of cells present in the defined area, inter-observer variation in the assessment of what is recognized by an individual pathologist as a mitotic figure, or the thickness of the section. These issues have surfaced recently as artificial intelligence (AI) tools begin to be used to count mitoses in digital whole slide images [16–18].

The *mitotic index* is the number of mitoses expressed as a percentage of the number of neoplastic cells present, identification of which can prove to challenge [19]. It does not depend on the area assessed, and so overcomes the issues of the area, intratumoral stroma, and cell size. This is not routinely done by pathologists who do not have time to count hundreds of cells in their routine clinical practice. However, it can be assessed using computational pathology methods on digital images, which may increase its utility in future studies.

The *mitotic rate* is defined as the rate at which cells are entering the mitotic (M) phase of the cell cycle, expressed as a percentage of the cells counted per hour. Whereas mitotic count and index are dependent on the duration of mitosis (which is variable), the mitotic rate is not affected, but assessment usually requires metaphase arrest or labeling methods in viable cells or tissue. It is therefore limited in its practical application for diagnostic purposes.

Both mitotic count and mitotic index depend on the recognition of mitoses. This can be assisted by immunohistochemistry for proteins expressed during mitosis, such as monoclonal mitotic protein-2 (MPM-2) [20] and phosphohistone H3 (PHH3) [21], though these stains may not correlate directly with morphological counts. While mitotic index and Ki-67 can both be used to measure cell proliferation, it is important to recognize that Ki-67 positively stains nuclei in G1, G2, or S phase, which is usually considerably longer and more variable than the M phase.

Mitotic activity varies within different regions of a tumor, so it is also important to decide whether a “hotspot” or “average” counting method should be employed. The recommended approach with the “hotspot” method is to first assess all slides from the tumor at relatively low power to find the region of the tumor containing more mitoses, so-called hotspots. After counting the mitoses in the initial high-power field with the highest number of mitoses within the “hotspot”, the count is extended to immediately adjacent non-overlapping fields until a specified area has been assessed. If no hotspot can be found and mitoses are sparse and/or randomly scattered throughout the tumor, then a representative mitosis is chosen and, beginning with that field, the count is then extended to immediately adjacent non-overlapping fields until the predetermined area has been assessed. With the “average counting method”, mitoses are assessed in randomly selected HPF and the average number of mitoses in a predefined area is expressed as a number per mm^2^. Modern computational systems have the capacity to do both across the whole slide and give a mean or median (for skewed data) depending on the requirements of the pathologist. However, when dealing with small biopsies, the use of mitotic counts is potentially problematic and prone to greater error due to sampling bias.

Pre-analytical factors can affect mitotic activity, particularly the time to fixation which may be prolonged if fresh tissue is required for other investigations, and ischemia within larger specimens. Some have identified a decline in the number of observable mitotic figures after delayed fixation [22]. However, delayed fixation may result in an increased mitotic count [23] as “the increase in mitotic figures in resection specimen and the significant shift towards metaphase figures is not due to a sampling artifact but reflects ongoing cell cycle activity in the resected tumor tissue due to fixation delay. The dwindling energy supply will eventually arrest tumor cells in metaphase, suggesting that rapidly fixed biopsy material better represents true tumor biology” [24], though cell stress may lead to an arrest in the M-phase [25].

Mitoses are usually detectable in multiple slices through cells, and tissue sections do vary in thickness; it is strictly true that the counting of mitoses per area of section is a volumetric assessment, and the thickness of the section should be considered [3]. However, in routine clinical practice utilizing good quality H&E stained sections of 3–5 μm in thickness, considerably less than the thickness of the cell, this is a relatively minor source of error and by convention is ignored. The recognition of mitoses is to some degree subjective. Nevertheless, the interobserver reproducibility of assessment of mitotic count by pathologists with varying levels of experience has been shown to be excellent in melanoma [4].

## Best practice for mitotic counts

There is evidence in melanoma that when mitoses are measured using a defined method and expressed as a number per mm^2^, there is excellent interobserver reproducibility in contrast to other studies in which mitoses have been expressed as number per HPF with poorer interobserver reproducibility [4, 26]. In the breast, a preliminary study proposed leveraging digital slides to count mitoses for phyllodes tumor grading over a larger tumor area of the entire tissue section than just 10 HPF. This could reduce potential interobserver selection bias, and would necessarily require reporting of mitotic counts per mm^2^ [27]. Strict criteria should be applied for accepting a mitotic figure in distinction from a pyknotic or apoptotic nucleus, and if there is uncertainty about a mitotic figure, it should not be counted.

In the fifth Edition WHO Classification of Tumors, SI units are used [28] and all mitotic counts must be reported per mm^2^, and if necessary qualified by the addition of a minimum area to count and whether an average across the tumor or hotspot counting method is to be used. It is acceptable to add “…count an area of at least ‘n’ mm^2^… in the area of highest mitotic activity” in brackets. Table 2 is included at the beginning of each WHO Blue Book volume for easy conversion of mitoses/mm^2^ to HPF based on knowing the diameter of the HPF in a pathologist’s own microscope. In some volumes where the use of HPF has commonly been used in the past as a diagnostic criterion, we have also provided the equivalent mitotic count expressed in HPF of defined size in the text to assist pathologists who are unfamiliar with these issues. Slides with 1 mm scales are widely available, making it a simple matter to check the size of HPF directly (Fig. 1) and these can also be scanned to check digital pathology systems.

**Table 2:** Approximate number of fields per 1 mm^2^ based on the field diameter and its corresponding area.

Field diameter (mm)	Field area (mm^2^)	Approximate number of fields per 1 mm^2^
0.40	0.126	8
0.41	0.132	8
0.42	0.138	7
0.43	0.145	7
0.44	0.152	7
0.45	0.159	6
0.46	0.166	6
0.47	0.173	6
0.48	0.181	6
0.49	0.188	5
0.50	0.196	5
0.51	0.204	5
0.52	0.212	5
0.53	0.221	5
0.54	0.229	4
0.55	0.237	4
0.56	0.246	4
0.57	0.255	4
0.58	0.264	4
0.59	0.273	4
0.60	0.283	4
0.61	0.292	3
0.62	0.302	3
0.63	0.312	3
0.64	0.322	3
0.65	0.332	3
0.66	0.342	3
0.67	0.352	3
0.68	0.363	3
0.69	0.374	3

Adapted from: WHO Classification of Tumors Editorial Board. Breast tumors. Lyon (France): International Agency for Research on Cancer; 2019. (WHO classification of tumors series, 5th ed.; vol. 2). https://publications.iarc.fr/581.

Unfortunately, if the diameter of the HPF used was not defined in the original research publication on a specific tumor, it is impossible to convert the data to mitoses per mm^2^ [29]. This leaves authors and editors in a difficult position, though a descriptive statement may be utilized. Such older studies often predate changes in treatment, and/or diagnosis, and probably need to be repeated. This is often the best solution, unless treatment has changed with an impact on survival, and can be done relatively simply if the slides and blocks are still available [30].

For a number of tumor types within the WHO Classification of Tumors [7, 8] where the mitotic count is a diagnostic criterion, the area to be assessed has been defined as the number of HPFs which must be assessed for mitoses by a pathologist, rather than the total area to be counted in mm^2^. This is erroneous but relatively unlikely to cause problems when many mitoses are present, though potentially problematic in small biopsies. The area to be counted may, in some instances, be based on published scientific studies that have evaluated the degree of variation in mitotic counts across sections, using cumulative mean or other methods to calculate the area required to get a reproducible measurement, but in other cases, it appears arbitrary.

**Fig. 2:**
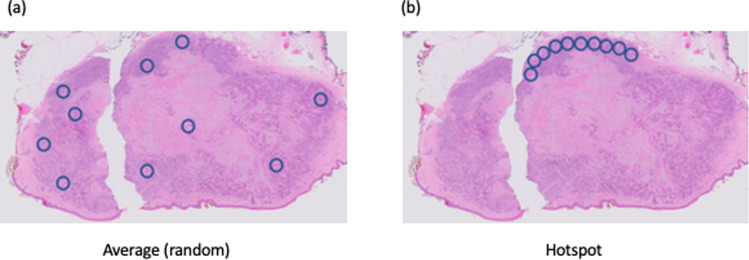
Hotspot counting based on square tiles or round microscope fields. Random counting (**a**) should use a randomization method to avoid bias, while hotspot counts (**b**) are usually linear or serpentine. It should be noted that contiguous round fields miss some areas of tumor which may contain mitoses. The method used should be clearly specified.

For the pathologist trying to determine the correct measurement, the problem is compounded by the requirement to assess hotspots (Fig. 2), or even the edge of tumors where there is the likelihood of interaction with the tumor microenvironment, and oxygen levels are likely to be best suited to neoplastic cell growth. This is of course something of Pandora’s box, but recent advances in digital pathology, make it a much less onerous task to return to this problem for those tumors where count per unit area is an important parameter determining malignancy or prognosis. It will be important to compare the methods wherever possible to avoid grade migration [31], though this seems relatively unlikely [16].

## Counting other features

The problem is not restricted to counting mitoses. Pathologists count other features within tumors and again, if these are expressed as a number per HPF, an error in their assessment is inevitable. In addition to mitoses, apoptotic bodies can be counted and expressed per mm^2^ [32]. Counting cells of particular types can be informative, and if this is done by morphology on H&E stained sections, then the counts should also be expressed per mm^2^. Follicular lymphoma is a case in point. In the 2017 fourth edition (revised) WHO Classification of Tumors [15], the count of centroblasts per HPF is used in grading. The field size is given as 0.159 mm^2^, but if this is ignored and a modern microscope with a field size of 0.23 mm^2^ used, then 30% more mitoses may be counted and the grade given may be higher than intended. As Table 2 shows, only 7 HPF and not 10, should be counted in this instance.

Microvessel density is a prognostic factor in a number of disparate tumor types, and has often been expressed as “vessels per HPF” with the inevitable consequence that results of studies cannot be compared unless the HPF is defined [1]. The best practice is to express these per mm^2^ as well, and this is compatible with digital systems that provide an automated assessment.

Tumor budding is an increasingly used prognostic factor in colorectal cancer and is assessed according to consensus guidelines [33], with definitions based on per mm^2^.

## Computational pathology

The issue of mitotic counts on digital images is particularly urgent for those pathology departments which are switching from using traditional light microscopes to digital pathology systems, as the latter does not have a round field of view (although a circular area of interest can usually be defined). There is no way of meeting any current guidance which specifies the use of HPF unless this is defined and thresholds converted accordingly. However, digital pathology systems do have the capacity to measure areas with considerable accuracy, without the requirement for calculation. For those using such systems, automated counting of cells identified by immunohistochemistry, or mitoses identified by deep learning methods, it is already possible to express these values per mm^2^ [16–18, 34]. This makes it even more important to avoid the use of non-standardized units and to switch to mitotic counts per mm^2^.

There is an increasing move to reporting pathology using digital images as the technology has improved significantly over the last 10 years, putting simple image analysis into the hands of pathologists during their routine work. Planimetric methods have been used for many years in research, but simple measurements of tumor depth and distance from margins of excision are now performed on digital images providing more accurate results. More sophisticated computational methods can also be applied to assess mitotic counts and the evidence is that these are as good or better than direct observation using the microscope, and are improving with time [17, 32, 35–37]. Comparative studies remain important to avoid potential grade migration [31]. The ability of pathologists to count mitoses accurately to provide prognostic information to clinicians has the potential to be greatly improved as a result.

In the future, digital pathology efforts may even facilitate the development of mitosis counting algorithms utilizing assessment of the entire tumor (even over multiple slides, levels, or biopsies). Furthermore, AI and machine learning may provide new opportunities to not only more precisely measure or even automate mitotic index measurements on specific tumor types, but they may also enable mitotic index and other digitally assessed tumor characteristics to be incorporated into new ways of classifying tumors.

## Recommendations for future work

Until digital pathology systems become more widely available throughout the world, there continues to be a need for pathologists to assess cell proliferation by counting mitoses for tumor diagnosis, grading, and prognosis. It is necessary to update knowledge in this area for a large number of different tumor types not only because an undefined HPF was often used in the original studies, but also because changes in treatment may mean that thresholds established long ago for mitotic counts (often as part of tumor grading) are no longer relevant when assessed with a modern microscope. Given that dichotomization of continuous variables is fraught with danger [38], it is arguably necessary to revisit such data, ideally in large studies or clinical trials, on a regular basis. As many trials now often require centralized pathology review, for which digital images are often obtained, there is some hope that this may be feasible. It is of course necessary for the pathologists involved in such studies and trials to define their measurements, using SI units, to allow corroboration of results across studies.

Implementation of standardized measurement is well advanced in many areas of pathology, but we hope that both scientific journal editors and the authors of pathology texts will insist on the use of mitoses per mm^2^, rather than per HPF.
